# Legal Management of Network Information Security Based on Embedded Real-Time Task Processing

**DOI:** 10.1155/2022/2379274

**Published:** 2022-09-09

**Authors:** Heju Bai

**Affiliations:** Law Teaching and Research Section, Hebei Petroleum University of Technology, Chengde, Hebei 067000, China

## Abstract

Nowadays, with the continuous advancement of globalization, Internet technology has made an unprecedented leap, which makes the amount of data on the Internet grow exponentially. Data are not only diverse and numerous, but also spread fast and of high value. Therefore, the Internet is entering the era of big data. However, the openness of information network itself, the immaturity of technology, and the imperfection of network security law make it vulnerable to network attacks, resulting in the disclosure of network users' information and the theft of their privacy data. The formulation of network information security law to protect network information security is of great significance for the development of multidomain network information security under the background of big data and ensuring users' online security. Firstly, this article introduces the basic information of embedded real-time task system, analyzes the factors of real-time Linux based on kernel preemption, interrupts mechanism and policy planning, and summarizes the process of some embedded real-time task processing. Integrating legal management, this article puts forward three elements of network security control mechanism, namely, network control personnel, environment, and technology. Taking these three elements as the starting point of the construction of information security control mechanism, this article provides the steps of using evaluation model to evaluate network information security. Finally, it identifies the problems existing in the legal management of network information security and formulates the corresponding strategies. By combining embedded technology and real-time task processing technology, this study applies it to the field of network information security legal management, to promote its development.

## 1. Introduction

Under the general trend of global information development, network information security has gradually become an important issue related to national security and social stability [1]. Based on this point, protecting network information security, carrying out information security management and control, and preventing network information data from being disturbed, damaged, leaked, and tampered have become the main work of the information security supervision department in the current environment [2]. In the information age, the scale of data flow information is too large, and the amount of information in various industries is increasing. Its transmission, processing, and storage mode is affected by big data technology, which makes the interaction and transmission of massive information possible and can break the time and space constraints to a certain extent [3]. Therefore, people increasingly rely on information systems, databases, and other technologies. Big data technology not only brings convenience to people, but also produces a series of problems, and has a higher demand for security performance [4]. For individuals, their privacy information is likely to be leaked under the action of network security vulnerabilities; for enterprises, their normal production process is inseparable from information security; for countries, network information security is a part of national security [5]. Therefore, network information security must be paid attention to and has become an important social problem [6]. The main task of the information control department is to ensure the security of network information. At present, although there are many types of network information security control facilities and related systems in China, they cannot effectively control the data security in the current big data environment, cannot play an effective role, and have defects to varying degrees [7]. This kind of management mode cannot meet the security requirements under the increasing demand of data transmission and management. Therefore, in order to ensure the high-speed transmission and exchange of big data information in the implementation of information security activities, it is necessary to establish a complete network information real-time task processing and verification system to ensure the security of network information [8]. Real-time embedded system not only contains the advantages of embedded system, but also meets the needs of high real-time. At present, embedded real-time systems can be seen in many industries. For example, intelligent control equipment, intelligent tools, and automatic control equipment require the embedded operating system to have high stability, reliability, and real-time, so that it can improve work efficiency based on the real-time task processing system network [9]. Finally, combined with various legal management, this study analyzes the current network security problems and limitations, hoping to help in the informatization of network security [10].

## 2. Related Work

The literature introduces the structure and characteristics of embedded Linux, summarizes the shortcomings and limitations of Linux in embedded applications, then analyzes the factors that hinder the real-time performance of Linux, such as kernel preemption, interrupt mechanism, scheduling strategy, and synchronous clock, studies and discusses the methods and programs of real-time Linux, and focuses on the basic principle and implementation mechanism of RT-Linux [11]. The literature puts forward the process of constructing and implementing campus network information security system. Based on the current situation of the network and focusing on the application core, this study puts forward the dynamic monitoring and emergency response of network information security, reduces the occurrence of security events, improves the system response speed, carries out source tracking and dynamic adjustment, and formulates the campus network information security scheme that meets the requirements of relevant national secondary standards [12]. The literature analyzes the current situation and information security problems of the school campus network and puts forward the detailed technology of physical security layer, network security layer, host security layer, application security layer, data security layer, and hierarchical security management, which meets the information security requirements of the current school network environment [13]. These requirements will provide a clear overall planning goal and direction of network information security for the school. This study constructs a new network information security evaluation index system niseis, takes the evaluation index as the influencing factor of network information security, and investigates the determination principle of the weight of the index system and the method of weight normalization. The exponential membership function model is used to assign a limit value to each level of a given index. Through unclear single factor evaluation, a comprehensive multilevel evaluation model is provided [14]. This study designs a network information evaluation system based on simulated vulnerability attack. By evaluating the system, the network security administrator can find the system vulnerabilities in time, take corresponding security protection measures, repair the system vulnerabilities, and ensure the safe operation of the network system [15].

## 3. Design of Embedded Real-Time Task Processing Principle

### 3.1. Structure of Embedded Linux System

**Figure 1:**
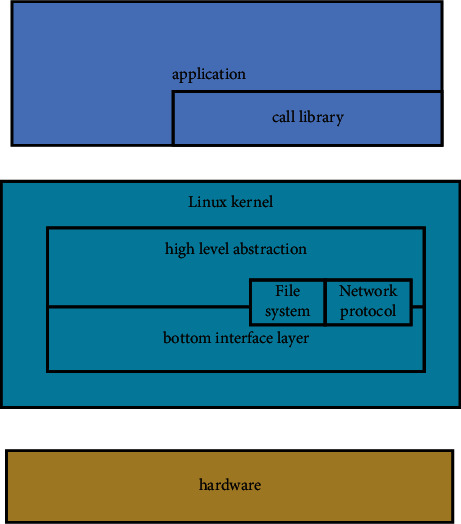
Structure of embedded Linux system.

Embedded Linux refers to a special Linux operating system with a capacity of only a few K or Mbytes that can be solidified on a memory chip or single chip microcomputer after miniaturization and standardization. It is generally suitable for specific applications, as shown in Figure 1.

Conventional embedded Linux system is usually composed of hardware, kernel, system call library, and top-level application software. The dependencies of these four layers are as follows: the top layer depends on the bottom layer, and each layer communicates with adjacent layers.

### 3.2. Measurement Standard of Real-Time System

Most embedded fields use real-time operating systems to judge whether an operating system is a real-time system, which is usually measured by the following indicators:

Deterministic: In a real-time operating system, the running time of its call can be predicted. This feature is deterministic. The maximum execution time of system call can be determined regardless of the load.

**Figure 2:**
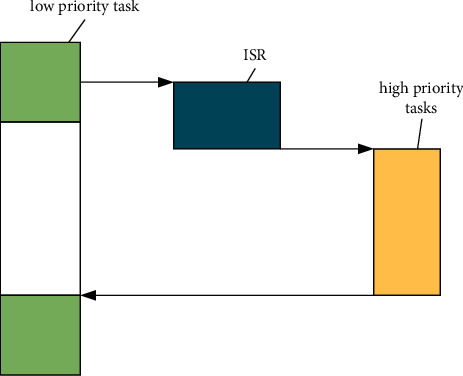
Preemptive kernel model.

Preemptive: The real-time operating system must be preemptive. In other words, the current task can be interrupted to allow another task to run, as shown in Figure 2.

Preemption can greatly improve the response of applications to asynchronous events. However, the core of the operating system is preemptive, which does not mean that task scheduling can be carried out at any time.

**Figure 3:**
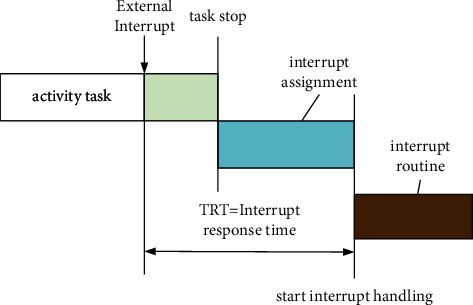
Interrupt response delay.

Figure 3 shows the interrupt response delay, which refers to the time from the occurrence of hardware interrupt to the execution of the first instruction in interrupt management.

Scheduling delay refers to the time between the time when an event causes a higher priority task to be ready and the time when the task starts running, that is, the time from preparation to running after the task is triggered. The delay is determined by the nonpreemptive time of the system and the specific algorithm.

### 3.3. Embedded Task Real-Time Scheduling Processing Method

The basic idea of real-time scheduling is to find the real-time task queue and compare the current exact time with the recovery task time *t*- > resume_ Time. If the current time exceeds the resume execution time, the resume execution time is *t* - > resume_ Time plus task interval *t*- > period (period cannot be 0. If period is 0, the resume execution time is *t* - > resume_time, and the assignment is infinite) The next running process is determined by the priority, so that the high priority process can seize the low-priority process.

**Figure 4:**
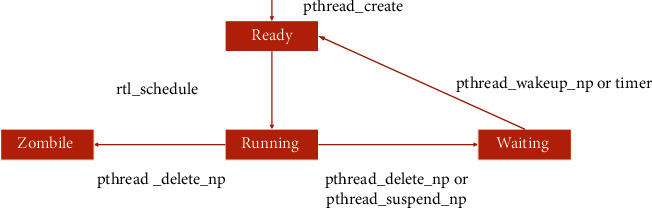
State transition of real-time tasks under RT-Linux.

RT-Linux scheduling directly supports real-time periodic tasks, and the running time and start time of each task can be set. Real-time aperiodic tasks are realized by defining interrupt service program. The scheduler treats Linux as a low-priority task so that it can be preempted by the highest priority task at any time. Non-real-time tasks can only run without real-time tasks. RT-Linux task scheduling divides the real-time process into four states (Ready, Zombile, Running, and Waiting), as shown in Figure 4.

In RT-Linux, the most important of all process-related functions is rtl_ schedule () function algorithm, which determines the real-time scheduling behavior of the process. For the real-time scheduling of *n* tasks, the following conditions need to be met to successfully schedule and ensure that each task does not exceed the set deadline.

(1) $$\begin{array}{c} \frac{C_{1}}{T_{1}}+\frac{C_{2}}{T_{2}}+\frac{C_{3}}{T_{3}}+\cdots \cdots \frac{C_{n}}{T_{n}}\leq n\left(2^{n}-1\right). \end{array}$$

RT-Linux scheduling mechanism also aims at modularization. Users can write their own scheduler according to their own needs, which can be realized through the loading module of the basic module. It allows users to easily test different scheduling strategies and algorithms to find the most suitable strategy for a specific application.

### 3.4. Relevant Code Design

The programming style and naming rules of this study are compatible with the single-core system, so the system can be compatible with the original single-core operating system. Users can select single-core system or SMP system configuration through macros. The key to complete the task scheduler is to execute the task scheduler, that is, the functions of the execution module, such as selecting backup tasks, selecting processor cores, scheduling execution.

SMP related macroconfiguration is as follows:

- #define OS_SMP_SUPPORT 1//macro configuration: 0 refers to single core system and 1 refers to SMP system
- #if OS_SMP_SUPPORT
- #define DSP_CORE_NUM 8//configure the number of processor cores. Currently, it is an 8-core processor
- #endif

The data structures related to SMP system task scheduling are defined as follows:

- #if OS_SMP_SUPPORT
- INT32U OSCtxSwCtr; //global context switching times
- INT8U OSIntNesting [DSP_CORE_NUM]; //number of interrupt nesting layers
- INT8U OSLockNesting [DSP_CORE_NUM]; //number of nested layers of multitask lock
- INT8U OSPrioCur [DSP_CORE_NUM]; //priority of currently running tasks on each core
- OS_TCB*∗*OSTCBCur [DSP_CORE_NUM]; //pointer to the current task control block of each core
- OS_TCB*∗*OSTCBCurOld;//pointer to the original running task on the currently scheduled core
- volatile INT32U OSIdleCtr[DSP_CORE_NUM]; //CPU idle count
- INT8U OSPrioHighRdy [DSP_CORE_NUM]; //backup task priority list
- #endif

The pseudocode to implement the selected backup task module is as follows. OS_ Sched_ Task () function finds all backup tasks, puts the backup task priority into the OSPrioHighRdy backup task priority list, and records and returns the SchTaskNum backup task number. Attachment: add hascpu member variable in TCB task control block to record whether the task occupies processor resources.

- int OS_Sched_Task (void)
- {
- //Copy ready table. Search in the copy ready table
- COPY_READYLIST;
- //Traverse N (number of processor cores) times to find all backup tasks
- for (int *j* = 0; *j* < DSP_CORE_NUM; *j*++){
- //Find the highest priority task in the copy ready table
- FIND_MAXTASK;
- //If the task currently has no processor resources, it will be added to the backup task list
- if (OSTCBPrioTbl[ copy_HighPrio ]- > hascpu = = 0){
- SchTaskNum ++;
- OSPrioHighRdy [SchTaskNum] = copy_HighPrio;
- }
- //Delete the task in the ready table (corresponding position 0)
- DELETE;
- }
- }
- return SchTaskNum;//return the number of backup tasks
- }

The pseudocode implemented by the basic processor module for selecting backup tasks is as follows. OS_ Sched_ The core() function selects the most appropriate processor core for the backup task list and returns the CPUid or coreid in the processor core. Attachment: taskPrio is the priority backup task, and LastCore is the processor core instruction assigned to the previous backup task. The affinity member variable is added to the TCB task control block to record the processor kernel that executed the backup task last time and to maximize the heat of the processor cache during scheduling.

- INT8U OS_Sched_Core (INT8U taskPrio, INT32U LastCore)
- {
- /*∗*Determines whether the processor core of the current scheduler is currently in idle state (the current task is empty, delayed or Pend).
- *∗*If it is idle, select the processor core to avoid using inter core interrupt to trigger*∗*task scheduling of the corresponding core*∗*/
- if (cupid ! = LastCore)
- if (the core (cupid) is free)
- return cpuid;
- /*∗* Judge whether the processor core of the last run of the task is idle. If it is idle, select the processor*∗*core to improve the cache hit rate in the processor core; *∗*/
- if (*j* ! = LastCore && *j* ! = NON_CORE)
- if (the core(*j*) is free)
- return *j*;
- //Select the task with the lowest priority to avoid redundancy
- for (*i* = 0; *i* < DSP_CORE_NUM; *i*++){
- SET the core that run the lowest prio task;
- }
- return coreId;//Returns the appropriate processor core assigned to the backup task
- }

The pseudocode implemented by the program execution module is given below. In SMP system, task scheduling is through calling task scheduler, that is, OS_ Sched function. According to OS_ Sched_ Task () backup task selection function and OS_ Sched_ The core () processor kernel selection function is called again.

- void OS_Sched (void)
- {
- for (*i* = 0; *i* ≤ num; *i*++){
- //Call processor core selection module
- schCpu = OS_Sched_Core (OSPrioHighRdy [i], schCpu);
- if (schCpu = = NON_CORE)
- break;
- else {
- //Set IPC inter core interrupt sending flag
- SET the core need interrupt;
- //Set the current nuclear task switching flag
- SET the task may be schedule in this core;
- //Send inter core task scheduling interrupt
- if (IPC_CPU! = 100)
- smp_send_reschedule (IPC_CPU);
- //Perform task switching
- if (aim_task_prio ! = OSPrioCur [cpuid])
- {
- OS_TASK_SW ();
- }
- }

## 4. Network Information Security Evaluation Method and Legal Management Strategy

### 4.1. Network Security Domain Planning and Topology Design

**Figure 5:**
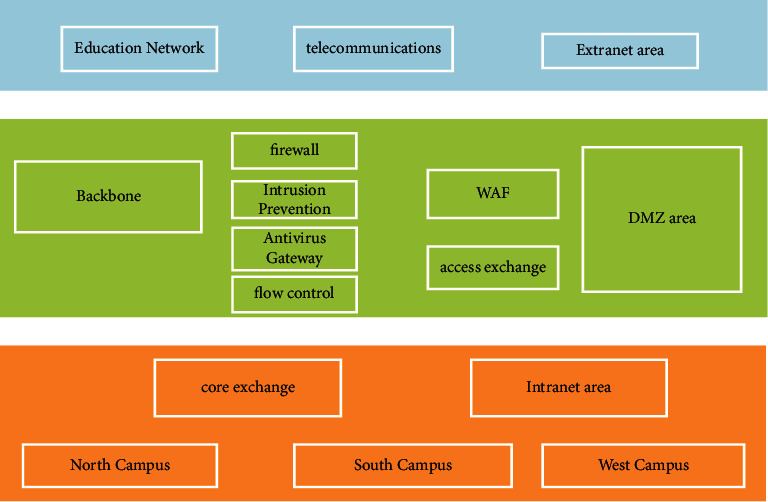
Network security domain planning topology.

Figure 5 shows the network security domain planning topology. Data disaster recovery technology is one of the most important technologies of network information security, and its development direction is networking and virtualization. By reasonably setting the backup mode, this technology can reduce the loss time of backup data to at least a few hours in case of various accidents. At the same time, through the operation of emergency response mode, the system can resume operation and prevent business interruption and other accidents caused by data loss.

**Figure 6:**
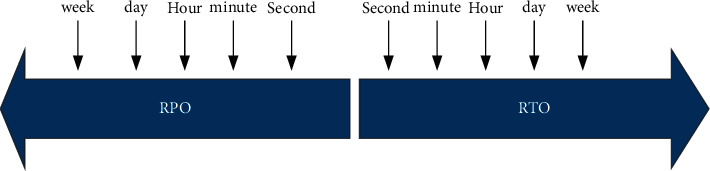
Schematic diagram of disaster recovery backup and recovery time nodes.

Data disaster recovery is divided into three levels: tape disaster recovery, data disaster recovery, and application disaster recovery. Values satisfying are RPO (data recovery point, tolerable data loss vector) and RTO (recovery time, tolerable recovery time), as shown in Figure 6.

### 4.2. Design of Network Information Security Evaluation Model

The problem of network security is not only a technical problem, but also a single problem, but a great system engineering. The realization of information security requires not only the use of technical means, but also many other means other than technology, such as the standardization of security standards, information management, and security management, which is accepted by many people. Pure technology cannot provide complete information security protection. More and more consensus is that security products alone cannot completely solve the problem of information security. Fuzzy comprehensive evaluation method is an important branch of fuzzy mathematics theory. The fuzzy transformation principle is used to comprehensively evaluate the objects considered. The method is mainly divided into two stages: the first step is the evaluation of one factor and the second step is the comprehensive evaluation of all factors.

Define the main factor layer index set as *U* = (U1, U2,..., Ui); the corresponding weight set is *W* = (W1, W2,..., Wi). Where uk (*k* = 1, 2, ..., *i*) represents the proportion of index Uk in U.

(2) $$\begin{array}{c} \overset{i}{\underset{k=1}{\sum }}w_{k}=1\text{.} \end{array}$$

Define the subfactor level indicator set Uk = (Uk1, Uk2,..., Ukm). The corresponding weight set is *Wk* = (*Wk*1, *Wk*2,..., *Wkm*). Where Wki (*i* = 1, 2, ..., m) represents the proportion of Uki index.

(3) $$\begin{array}{c} \overset{i}{\underset{k=1}{\sum }}w_{\text{ki}}=1\text{.} \end{array}$$

The fuzzy evaluation matrix from Uk to U is defined as Rk:

(4) $$\begin{array}{c} R_{k}=\left(\begin{array}{cccc} r_{11} & r_{12} & \cdots & r_{1n}\\ r_{21} & r_{22} & \cdots & r_{2n}\\ \cdots & \cdots & \cdots & \cdots \\ r_{m1} & r_{m2} & \cdots & r_{\text{mn}} \end{array}\right)\text{,} \end{array}$$

where rij (*i* = 1, 2, ..., *m*; *j* = 1, 2, ..., n) represents the grade index of the subfactor Uki, and comments on the attribution degree of Vj for the j-th grade, that is, rij represents the subindex. Uki analysis belongs to the membership of the fuzzy set specified in the j-level comment. The value of rij is determined as follows: the designed expert questionnaire is distributed to several experts, who decide the items to be weighed according to various influencing factors. Then, the results of expert evaluation are statistically compiled to obtain Vi1 V1 comments, Vi2 V2 comments, Vin VN level comments of Uki index, then for *i* = 1, 2,…, m, there are:

(5) $$\begin{array}{c} r_{\text{ij}}=\frac{w_{\text{ij}}}{\overset{n}{\underset{i=1}{\sum }}W_{\text{ij}}\left(j=1,2,\ldots ,n\right)}\text{.} \end{array}$$

Therefore, Uk is a fuzzy mapping from the sub factor layer to the target layer.

For this purpose, in the following, a boundary value is assigned to each level of a given index.

When *j* = 1, the membership function is:

(6) $$\begin{array}{c} u_{\text{vj}}\left(u_{i}\right)=\left\{\begin{array}{cc} 1\text{,} & u_{i}\geq d_{j}\text{,}\\ \frac{\left(u_{i}-d_{j+1}\right)}{d_{j}-d_{j+1}}\text{,} & d_{j+1}\leq u_{i}<d_{j}\text{,}\\ 0\text{,} & u_{i}<d_{j+1}\text{.} \end{array}\right. \end{array}$$

When *j* = 2,3, ..., n-1, the membership function is:

(7) $$\begin{array}{c} u_{\text{vj}}\left(u_{i}\right)=\left\{\begin{array}{cc} \frac{\left(u_{i}-d_{j+1}\right)}{d_{j}-d_{j+1}}\text{,} & d_{j+1}\leq u_{i}<d_{j}\text{,}\\ \frac{d_{j-1}-u_{i}}{d_{j-1}-d_{j_{i}}}\text{,} & d_{j}\leq u_{i}<d_{j-1}\text{,}\\ 0\text{,} & u_{i}\geq d_{j-1}or\;u_{i}<d_{j+1}\text{,} \end{array}\right. \end{array}$$

(8) $$\begin{array}{c} u_{\text{vj}}\left(u_{i}\right)=\left\{\begin{array}{cc} 0\text{,} & u_{i}\geq d_{j-1}\text{,}\\ \frac{d_{j-1}-u_{i}}{d_{j-1}-d_{j}}\text{,} & d_{j}\leq u_{i}<d_{j-1}\text{,}\\ 1\text{,} & u_{i}<d_{j+1}\text{.} \end{array}\right. \end{array}$$

In the fuzzy comprehensive evaluation, first evaluate from a group of U factors and determine the attribution degree of the evaluation object in each element of the group of comments. The evaluation result is expressed as *R* by a fuzzy set, which is a fuzzy set in the comment set V. The set of n-Factor evaluation is called the total evaluation matrix.

(9) $$\begin{array}{c} R_{1}=\left[\begin{array}{cccc} r_{11} & r_{12} & \ldots & r_{1n}\\ r_{21} & \ldots & \ldots & \ldots \\ \ldots & \ldots & \ldots & \ldots \\ r_{m1} & r_{m2} & \ldots & r_{\text{mn}} \end{array}\right]\text{,} \end{array}$$

(10) $$\begin{array}{c} R_{2}=\left[\begin{array}{cccc} r_{11} & r_{12} & \ldots & r_{1n}\\ r_{21} & \ldots & \ldots & \cdots \\ \cdots & \cdots & \ldots & \cdots \\ r_{m1} & r_{m2} & \ldots & r_{\text{mn}} \end{array}\right]\text{,} \end{array}$$

(11) $$\begin{array}{c} R_{m}=\left[\begin{array}{cccc} r_{11} & r_{12} & \ldots & r_{1n}\\ r_{21} & \ldots & \ldots & \cdots \\ \cdots & \ldots & \ldots & \cdots \\ r_{m1} & r_{m2} & \ldots & r_{\text{mn}} \end{array}\right]\text{,} \end{array}$$

where rmn = *R* (uivi), expressed as the degree of membership of rmn to the evaluation vi.

Firstly, the evaluation matrix Rk of each subfactor index Uki is subjected to fuzzy matrix operation, and the membership vector Bk of the main factor index Uk in the comment set F is taken:

(12) $$\begin{array}{c} B=W\circ R=\left(B_{1},B_{2},\ldots \ldots B_{i}\right)\text{.} \end{array}$$

Wk was previously determined by analytic hierarchy process, and Rk was also determined as described above.

(13) $$\begin{array}{c} R=\left(\begin{array}{c} B_{1}\\ \vdots \\ B_{i} \end{array}\right)\left(\begin{array}{cccc} b_{11} & b_{12} & \cdots & b_{1n}\\ b_{21} & b_{22} & \cdots & b_{2n}\\ \cdots & \cdots & \cdots & \cdots \\ b_{i1} & b_{i2} & \cdots & b_{\text{in}} \end{array}\right)\text{.} \end{array}$$

Then calculate the fuzzy matrix of *R* to obtain the membership vector B of the target layer index U to the comment set F

(14) $$\begin{array}{c} B=W\;R=W\left(w_{1},w_{2},\cdots ,w_{i}\right)\left(\begin{array}{c} B_{1}\\ B_{2}\\ \vdots \\ B_{i} \end{array}\right)=\left(b_{1},b_{2},\cdots ,b_{n}\right)\text{.} \end{array}$$

When∑_*i*=1_^*n*^*b*_*j*_ is not equal to 1, it can be normalized.

Therefore, the general fuzzy comprehensive evaluation model of network information security is:

(15) $$\begin{array}{c} B=W\times R=W\times \left(\begin{array}{c} B_{1}\\ B_{2}\\ \vdots \\ B_{i} \end{array}\right)=W\times \left(\begin{array}{c} W_{1}\times R_{1}\\ W_{2}\times R_{2}\\ \vdots \\ W_{i}\times R_{i} \end{array}\right)\text{.} \end{array}$$

According to fuzzy comprehensive evaluation:

(16) $$\begin{array}{c} B=W\circ R=\left(\begin{array}{c} B_{1},B_{2},\ldots \ldots B_{i} \end{array}\right)\text{.} \end{array}$$

According to the principle of maximum membership degree, the corresponding grade of maximum Bi is the result of evaluation.

From the perspective of personnel, environment, and technology, this study designs and constructs a multilevel and multi-index network information security evaluation index, as shown in Table 1.

For the layer sorting results of each index layer, the total weight of each index can be obtained by calculating the results in the order of layers. The sorting results of the total hierarchy are shown in Table 2:

Therefore, from the overall ranking of the hierarchy, we can see that the main problems of network information security mainly focus on the following aspects: antivirus measures, security system, antihacker security measures, and user identification measures. First of all, because the current viruses and hacker software attack and destroy the network in different ways, the focus of network security investment is to deal with viruses and hackers. Secondly, the information security system of the network system is not perfect, because many people do not abide by the rules and have no rules to follow, so the network crime is in a state of disorder.

### 4.3. Problems in Legal Management of Network Information Security

At present, even if laws and regulations impose obligations on the responsible person and adopt privacy policies for the network information they collect to ensure that the network information is not disclosed, the responsibility and punishment for further violations are rarely mentioned. Even if the relevant liability is established, due to the influence of the thought of “heavy punishment over the people,” although heavy punishment and criminal punishment are given to serious crimes, civil and reputation relief compensation is still a blind spot. The main body of the information network is still unable to obtain sufficient relief, which will reduce the cost of illegal crime and inevitably destroy the security of the information network. With the continuous development of social economy and the increasing maturity of information network technology, the commercial value of information has increased infinitely. Many legal persons and other organizational units specializing in network information services have emerged in the market. Users only need to pay a part of the fee to extract a large number of other people's information and relevant materials from these individuals or organizations, and the cases of illegal profit-making are also increasing year by year. Therefore, the establishment of a perfect civil relief and compensation mechanism is conducive to the security of information network subjects, and it is also an effective way to sanction network criminals to obtain illegal funds.

Ordinary people may not know how network information is leaked, and the most unsafe link is the government administrative department. Restricting public power according to law has become an urgent problem to be solved. At present, the law rarely mentions that the government and other enterprises and institutions must bear legal responsibility for the leakage of network information. In the decision of the Standing Committee of the National People's Congress on strengthening the security protection of network information, there are few provisions on the disclosure of network information of public institutions, and state organs are not among them. Even if there are regulations, the management department cannot be determined, so the corresponding punishment will not be mentioned. Its principle is relatively strong, but its execution is weak. In practice, government agencies are often seen as violating the privacy and security of citizens' online information. In order to perform its duties, the government must violate the network information security at a specific time, and its behavior is protected by law. Therefore, the infringement in this case does not belong to illegal infringement. The law on the protection of network information security has not been made public and is still in the stage of design and consultation. One of the important reasons is that it cannot effectively prevent government administrative departments from using public power and ensure that network information security does not infringe on public power, which will involve many interests. Therefore, the promulgation period of the network information security protection law has been extended indefinitely.

### 4.4. Improvement Strategy of Legal Management of Network Information Security

Perfecting the legislative system is the basis for establishing the standards and procedures for the identification of criminal acts. From the current legislative situation in China, the legislation of network information security protection has not yet formed a complete system. The author believes that in order to obtain effective protection of network information security, we must start from the following aspects. First of all, the restrictive role of the constitution is fundamental. Based on the constitution, the effective protection of network information security must be clearly stipulated. Even if no explicit provisions are adopted, this basic legislative thought should be limited and specific laws and regulations should be fully developed and supplemented, such as the general principles of civil law or the tort liability law; Secondly, the formulation of special laws and regulations can effectively avoid various privacy risks, make the protection of network information more secure, reduce the risk of information leakage, make users' online environment more secure, and promote the realization of public interest needs; the last is to clarify the definition of network security protection in civil law. The civil law defines the privacy right of ordinary individuals and makes additional provisions on the protection right of network information security, such as freedom of communication, freedom of personal life, and so on. In addition, the content and scope of public interest and the reasonable public interest in laws and regulations must be clear.

In order to effectively solve the legal problems of protecting network information security around the network environment, we must clarify the definition and responsibility of the subject and object of crime. Around the network, the subject of crime is government departments, legal persons, and other organizations, as well as individual network users, while the object of crime is network operators and service providers, as well as specific criminal individuals and organizations. Once the subject and object of the crime are clarified, the legal responsibility they should bear can be clarified in order to effectively protect the security of network information. Clarifying the division of responsibility of criminal participants is an important work of law enforcement. The division of legal responsibility is to measure the subject and proportion of responsibility from the perspective of law enforcement, so as to increase the role of protecting network information security.

In order to prevent the leakage of network information, we must strengthen sentencing and management, so as to create a good network environment and social environment and ensure the security of network information. A special network security protection platform shall be established and implemented by relevant units simultaneously. On this basis, a special department shall be established to manage and supervise the business development of the whole organization. Once any violation is found, it shall be handled in time. According to the scale of the plan, violations will be handled appropriately to maintain the security of network information. At the same time, the confidentiality of specific network information shall be disclosed. The media can guide, warn, and educate the public on morality and social values by exposing their crimes, violations of law and discipline, and other violations in real time. Similarly, it is also necessary to establish systems related to network information security protection. There will be other legislative provisions for the protection and control of information security in the network, so as to realize the law to follow. The Constitution clearly stipulates those citizens have the right to criticize and make suggestions to state organs and personnel, but there are no detailed guidelines for their operation. Therefore, the judiciary must fully legislate and strictly implement it in order to formulate relevant protection and prohibition rules. With the above content, we can increase the punishment for violation of network information security, ensure network information security and be protected by law, and enable users to spontaneously bear the same social responsibility while enjoying their rights.

## 5. Conclusion

With the continuous progress of computer network technology, people can access and use information in a wide range in a short time. However, due to the openness, ease of use and sharing of the network, the lack of network security technology, and the imperfection of network security law, network information is constantly threatened by leakage, which seriously affects the security of network user information. In recent years, there have been constant disputes over information disclosure caused by network insecurity events such as human flesh search, advertising, information fraud, and SMS harassment. Therefore, this study first puts forward the embedded system for real-time processing of network information tasks and puts forward the topology scheme of network security domain planning, standard real-time measurement system, the structure and implementation method of embedded Linux system, including embedded task processing, real-time scheduling, and relevant rules. In the process of discussing the current network security law, this study puts forward the existing shortcomings and improvement strategies. This study hopes to help maintain network information security and provide a safe Internet environment for Internet users.

## Figures and Tables

**Table 1:** Evaluation index system of network information security control mechanism (*G*).

Primary index T	Secondary index C	Tertiary indicators P
Personnel T1	Manager C1	Technical capabilities P1
		In-job staff safety literary P2
		Professional P3
	Network information service provider C2	Information service provider's security behavior P4
		Information service provider's security responsibility P5
	User C3	User safety education P6
		User security awareness P7
		User's self-management P8

Environment T2	Network facility C4	P9 facilities P9
		Bearing capacity P10
	Regulations, policies C5	Network information security standard P11
		Network information security legislation P12
		Network information security behavior specification P13

Technology T3	Encryption technology C6	Communication encryption technology P14
		Storage encryption technology P15
	Safety monitoring technology C7	Database access monitor P16
		System access monitor P17
		Operating system access monitoring P18
	Firewall technology C8	User access permissions P19
		Anti-attack software P20
		Anti-refusal service attack P21
		User identification P22
	Safety audit technology C9	Application log audit P23
		Intrusion detection monitoring audit P24
		Operation log audit P25
		Antivirus upgrade audit P26
		Database log audit P27

**Table 2:** Total ranking of levels.

Target layer	Standard layer	Indicator layer	Weights	Sort
Network information security evaluation index system	Safety technical measures 0.28	Safety audit function	0.162	0.0469
		System monitoring log	0.101	0.0293
		Anti-third-party invading facility	0.253	0.0732
		System backup measures	0.071	0.0205
	Safety system 0.32	Emergency accident handling plan	0.337	0.1181
		Emergency rules and regulations	0.336	0.111
		Organization	0.336	0.111
		Antivirus measures	0.424	0.123
	Network communication safety 0.11	Important system backup measures	0.111	0.0133
		Safety encryption measures	0.162	0.0194
		Access monitoring measures	0.263	0.0315
		Safety audit measures	0.475	0.057
	Physical safety 0.10	Media security	0.626	0.0626
		Environment safety	0.141	0.0141
		Equipment security	0.242	0.0242
	System security 0.15	User information backup measures	0.081	0.0129
		System antifailure measures	0.152	0.0242
		User identification measures	0.384	0.0687
		Database and system status audit facilities	0.04	0.0646
		Database access and operating system monitoring measures	0.035	0.0566

## Data Availability

The data used to support the findings of this study are available from the corresponding author upon request.
